# Hyperosmolar Therapy in Severe Traumatic Brain Injury: A Survey of Emergency Physicians from a Large Canadian Province

**DOI:** 10.1371/journal.pone.0095778

**Published:** 2014-04-22

**Authors:** Elyse Berger Pelletier, Marcel Émond, François Lauzier, Martin Savard, Alexis F. Turgeon

**Affiliations:** 1 Research Center of the Centre Hospitalier Universitaire (CHU) de Québec, Axe Santé des populations et pratiques optimales en santé (Population Health and Optimal health practices Research Unit), Traumatologie - Urgence - Soins Intensifs (Trauma – Emergency – Critical Care Medicine), CHU de Québec (Hôpital de l′Enfant-Jésus), Université Laval, Québec City, Québec, Canada; 2 Department of Family and Emergency Medicine, Université Laval, Québec City, Québec, Canada; 3 Division of Critical Care, Department of Anesthesiology, Université Laval, Québec City, Québec, Canada; 4 Department of Medicine, Université Laval, Québec City, Québec, Canad; 5 Department of Neurological Sciences, Université Laval, Québec City, Québec, Canada; Weill Cornell Medical College, United States of America

## Abstract

**Introduction:**

Worldwide, severe traumatic brain injury is a frequent pathology and is associated with high morbidity and mortality. Mannitol and hypertonic saline are therapeutic options for intracranial hypertension occurring in the acute phase of care. However, current practices of emergency physicians are unknown.

**Methods:**

We conducted a self-administered survey of emergency physicians in the province of Québec, Canada, to understand their attitudes surrounding the use of hyperosmolar solutions in patients with severe traumatic brain injury. Using information from a systematic review of hypertonic saline solutions and experts' opinion, we developed a questionnaire following a systematic approach (items generation and reduction). We tested the questionnaire for face and content validity, and test-retest reliability. Physicians were identified through the department head of each eligible level I and II trauma centers. We administered the survey using a web-based interface and planned email reminders.

**Results:**

We received 210 questionnaires out of 429 potentials respondents (response rate 49%). Most respondents worked in level II trauma centers (69%). Fifty-three percent (53%) of emergency physicians stated using hypertonic saline to treat severe traumatic brain injury. Most reported using hyperosmolar therapy in the presence of severe traumatic brain injury and unilateral reactive mydriasis, midline shift or cistern compression on brain computed tomography.

**Conclusion:**

Hyperosmolar therapy is believed being broadly used by emergency physicians in Quebec following severe traumatic brain injury. Despite the absence of clinical practice guidelines promoting the use of hypertonic saline, a majority of them said to use these solutions in specific clinical situations.

## Introduction

Severe traumatic brain injury is frequent and associated with significant mortality and morbidity [1], [2], [3], [4]. The development of intracranial hypertension is the mechanism by which secondary cerebral injury can occur and worsen patients' outcome [5], [6]. Many therapeutic modalities are available and used in clinical practice, but few are lifesaving in the emergency room [7]. As part of the recommendations of the Brain Trauma Foundation guidelines for the management of patients with severe traumatic brain injury, mannitol is often used by emergency physicians to treat presumed increased intracranial pressure [8], [9]. By generating an osmolar gradient, mannitol helps shift water from the brain cells and interstitium into the intravascular space, and thus decreases intracranial pressure [10]. However, considering the potential side effects of mannitol [11], [12], the use of other hyperosmolar solutions such as hypertonic saline solutions has gained popularity over the last decade [13], [14], [15]. Initially used in a military setting to minimize weight carriage of resuscitation fluids, it was also suggested in patients with severe traumatic brain injury as hyperosmolar therapy especially in presence of hemodynamic instability [16], [17]. As opposed, mannitol being a diuretics, the maintenance of the fluid balance and the hemodynamic stability may sometimes be challenging. Despite the absence of recommendation promoting their use, hypertonic saline solutions are utilized more commonly by physicians as a first or second line agent in the intensive care unit [2], [13], [15]. Very little information is however currently available on the use hypertonic saline solutions among emergency physicians. Considering the expanding use of these solutions in Canada [13] despite limited evidence of clinical benefit, along with current guidelines suggesting minimizing the use of hyperosmolar therapies prior to monitoring intracranial pressure, a better understanding of their utilization is needed.

Hence, we conducted a self-administered electronic survey of Québec emergency physicians, the second largest Canadian province, to understand their perception and opinion towards the use of hyperosmolar solutions in patients with severe traumatic brain injury.

## Methods

### Ethics statement

The study was approved by the Research Ethics Board of the Centre Hospitalier *Affilié* Universitaire de Québec (CH*A*) (Hôpital de l′Enfant-Jésus), Québec City, Québec, Canada.

### Study population

We conducted a self-administered cross-sectional survey of emergency physicians from the province of Québec, Canada, that are involved in the care of severe traumatic brain injury patients. The province of Québec is the second largest Canadian province with a population of more than 8 millions inhabitants. Emergency physicians working in level I (comprehensive trauma services with academic leadership, teaching and research programs) and II trauma (comprehensive trauma services without academic and research programs) centers in the province of Québec where an estimated mean of at least one adult patient with severe traumatic brain injury per month consulting in the emergency room were considered eligible, since we wanted to survey emergency physicians with a minimum of experience in treating severe head injury patients. We screened the *Registre des traumatismes du Québec* (Québec Trauma Registry) to select the eligible trauma centers. Emergency physicians from three level I and 11 level II trauma centers were thus considered. Potential respondents were identified through department heads of the targeted centers. All department heads accepted to share their members' contact information with the exception of one level II trauma center. Physicians from this center were thus not part of this study.

### Questionnaire development

The development of the questionnaire was done following recent recommendations for self-administered survey methodology [18], [19] similar to a previous survey conducted by our team [20]. First, we conducted a literature review on prognostic factors and therapeutic options in severe traumatic brain injury during the acute phase of care. Then, we conducted a systematic review on the use of hyperosmolar solutions in patients with severe traumatic brain injury (unpublished data). Afterwards, seven clinical experts from different medical specialties (emergency medicine, critical care medicine and neurology) generated a list items thought to be relevant to the administration of hyperosmolar solutions and have been shown or thought to influence the decision-making process. We grouped items into 2 categories: predictors (e.g., comorbidities, age) and factors (e.g., data from the scene). Lastly, the group of experts did several items rating iterations to ensure the inclusion of the most relevant items. The survey questionnaire was designed using the definitive items.

### Questionnaire key sections

#### 1) Perceived use of hyperosmolar solutions

Using a 5-point Likert scale (never, rarely, sometimes, often, always), we evaluated the perceived utilization of mannitol and hypertonic saline solutions in the management of presumed intracranial hypertension in patients with severe traumatic brain injury.

#### 2) Factors modifying the intention to use hyperosmolar solutions

Using a clinical scenario and a 7-point Likert scale (never, rarely, sometimes, often, very often, almost always, always), we asked emergency physicians if clinical or radiological findings would change their intention to use hyperosmolar solutions. The baseline scenario described a 35 year-old male with severe blunt head injury, a GCS of 3, normal vital signs and without other traumatic injuries (Appendix S1). We modified the scenario according to different characteristics associated with severity (e.g. age, comorbidities, computed tomography [CT] scan results) and repeated the same questions. Eleven modifications of the baseline scenario were therefore created. For this section, respondents did not have to mention which hyperosmolar solution they would use.

#### 3) Facilitators or barriers for the use of hyperosmolar solutions

We presented a series of choices to examine which factors would influence positively (e.g., studies supporting the use of mannitol to reduce mortality) or negatively (e.g., hypertonic saline unavailable in their emergency room) their intention to use mannitol and/or hypertonic saline.

### Questionnaire testing and validation

Following the development of the questionnaire, it was pilot-tested by all the participating experts to assess its accessibility. Then, sensitivity testing using a standardized form was performed to identify major omissions and evaluate the relevance of our survey.

Following sensitivity testing, we conducted a test-retest reliability assessment by asking 10 emergency medicine residents to complete the survey at different time intervals (two weeks apart). We evaluated the reliability using kappa statistics [21]. The questionnaire was developed in French given that the vast majority of emergency physicians in Québec are primarily French speaking or bilingual (English/French). The questionnaire is presented in appendix S1.

### Questionnaire administration

We administered the questionnaire using a Web-based software (Surveymonkey, www.surveymonkey.com). Prior to the survey administration, all potential respondents received an e-mail inviting them to complete the survey. No incentive was used. Every two weeks, an e-mail reminder was sent to all non-respondents. After two months, a paper version of the questionnaire was mailed to the non-respondents along with a pre-stamped return envelope. A postcard was sent 4 weeks after the initial mailing for those who had not returned the questionnaire.

### Sample size

We estimated the total number of potential respondents to be approximately 400. With an anticipated response rate around 50% based on recent published surveys in this population, we anticipated receiving 200 completed questionnaires. Conservatively, this sample size provided power to generate 95% confidence interval of 7% around a response item, based on the assumption that the true proportion is 50%.

### Statistical analyses

We used Cohen kappa statistics to analyze the questionnaire's reliability; more than 80% of the kappa scores were >0.40 thus representing moderate to good agreement [18]. A questionnaire was considered completed when over 80% of the questions were answered [22]. We summarized survey responses using descriptive statistics (proportions with 95% confidence intervals). When deemed appropriate, we pooled categories of Likert scales to present the results in a meaningful manner.

## Results

**Figure 1:**
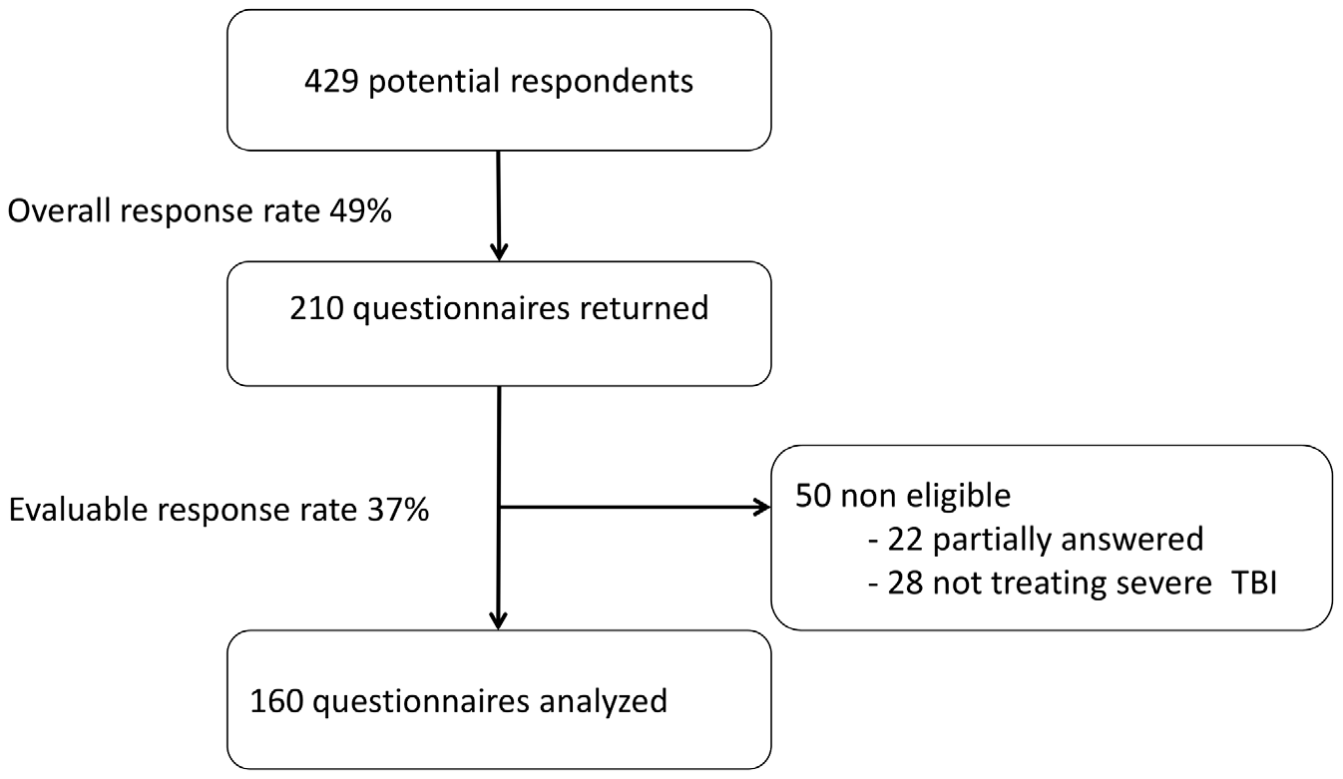
Flow diagram of participants.

We identified 429 potential respondents (Figure 1). We received 210 questionnaires (response rate 49%). Twenty-two questionnaires were partially answered (<80%) and thus not analyzed. Twenty-eight respondents said that they did not care for patients with severe traumatic brain injury and were considered ineligible. One hundred and sixty questionnaires were included the final analysis (Figure 1).

### Respondent characteristics

Most respondents were full-time emergency physicians (>12 shifts per month) (n = 98, 61%) with family medicine training (n = 135, 84%) and had between 1 to 10 years of experience (n = 92, 58%). Half of the respondents reported having access to a neurosurgeon (n = 79, 49%) (Table 1).

**Table 1:** Respondents' demographic characteristics.

		N (%)
Baseline training	Family medicine	62 (38.8)
	Family medicine with emergency medicine training	73 (45.6)
	Emergency medicine specialist	25 (15.6)
Years of experience	Less than one	8 (5.0)
	1 to 5	47 (29.4)
	6 to 10	45 (28.1)
	11 to 20	34 (21.3)
	More than 20	26 (16.3)
Numbers of emergency shifts per months	Less than 4	4 (2.5)
	5 to 8	18 (11.3)
	9 to 11	40 (25.0)
	More than 12	98 (61.3)
Type of trauma centre	Level one	49 (30.6)
	Level two	111 (69.4)
Neurosurgeon available in house		79 (49.40)

### Use of hyperosmolar solutions

**Figure 2:**
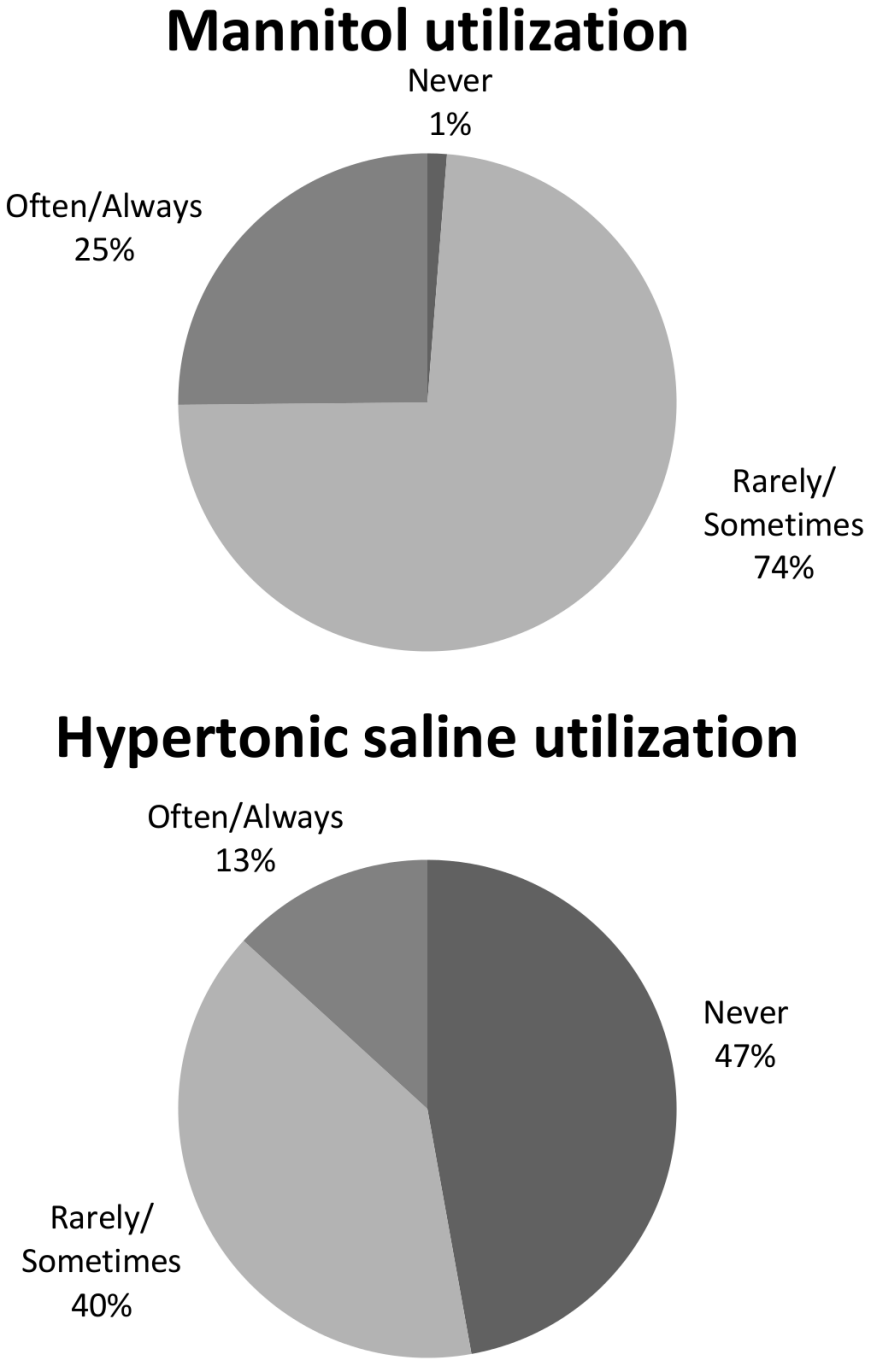
Perceived utilization of hyperosmolar solutions.

Ninety-nine percent of the respondents reported using mannitol, 74% (95% confidence intervals [Cl] 66–80%) reported using it rarely or sometimes, and 25% (95% CI 18–32%) often or always (Figure 2). In regards to the use of hypertonic saline solutions, 53% (95%Cl 45–60%) of respondents reported using it regularly, 40% (95%CI 32–48%) reported rarely or sometimes and 13% (95%Cl 9–19%) reported using hypertonic saline solutions often or always.

### Factors influencing the intention to use hyperosmolar solutions

**Figure 3:**
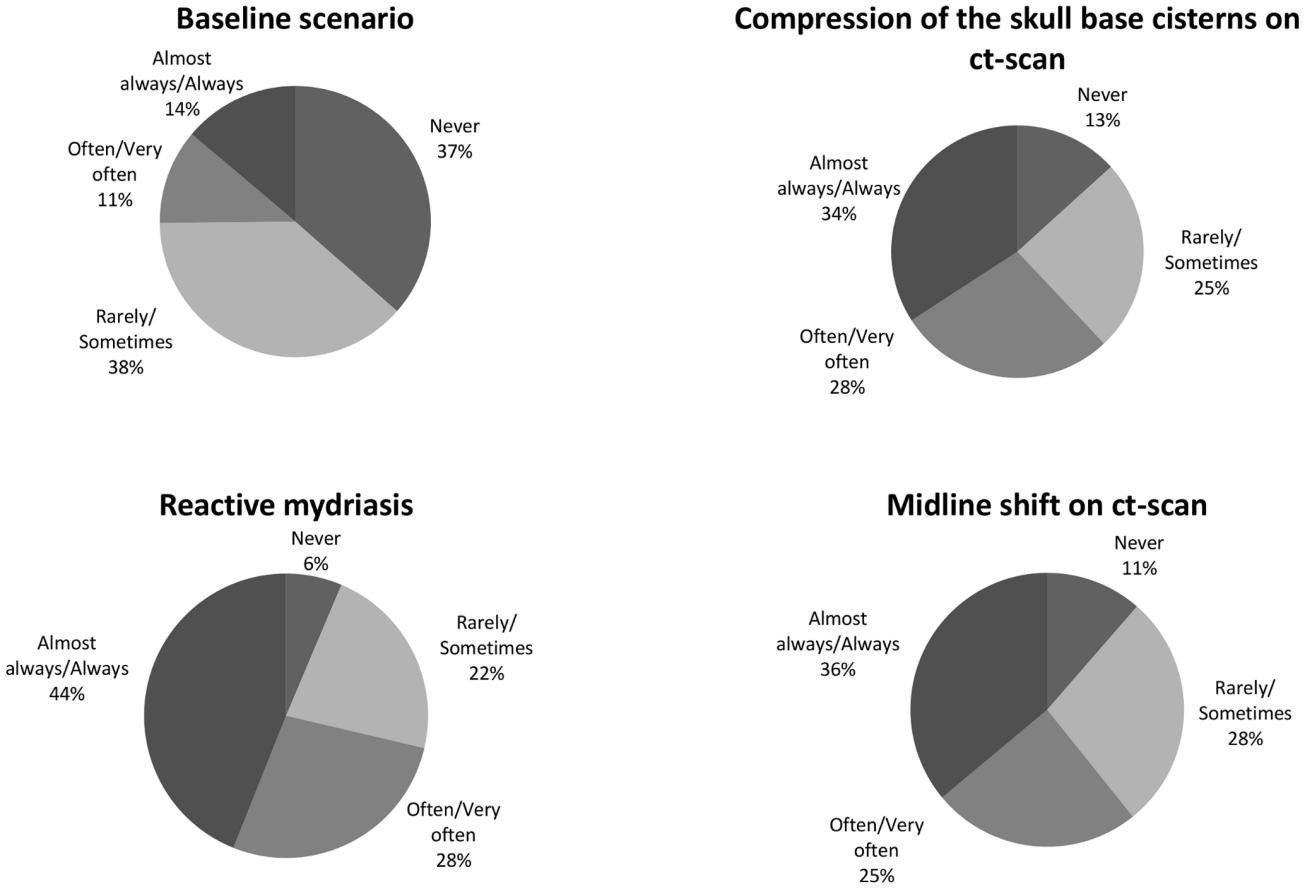
Factors influencing the intention of use of hyperosmolar solutions.

In the baseline scenario, 58 respondents answered they would never use any hyperosmolar solutions (36%, 95%Cl 29–44%), 61 answered that they would use them rarely or sometimes (38%, 95%Cl 31–46%), 18 replied that they would use hyperosmolar solutions often or very often (11%, 95%Cl 7–17%) and 22 stated that they would almost always or always (14%, 95%Cl 9–20%) use them. These answers varied depending on scenario modifications. Indeed, most respondents said that they would use hyperosmolar solutions always or almost always in the presence of a reactive dilated pupil (44%, 95%Cl 36–52%), compression of the basal cisterns (34%, 95%Cl 27–42%) or a mid-line shift on CT-scan (36%, 95%Cl 29–44%) (Figure 3). However, regardless of the presented scenario, a few respondents (6–13%) answered that they would “never” use hyperosmolar solutions.

### Facilitators and barriers to the use of hyperosmolar solutions

#### Facilitators

**Figure 4:**
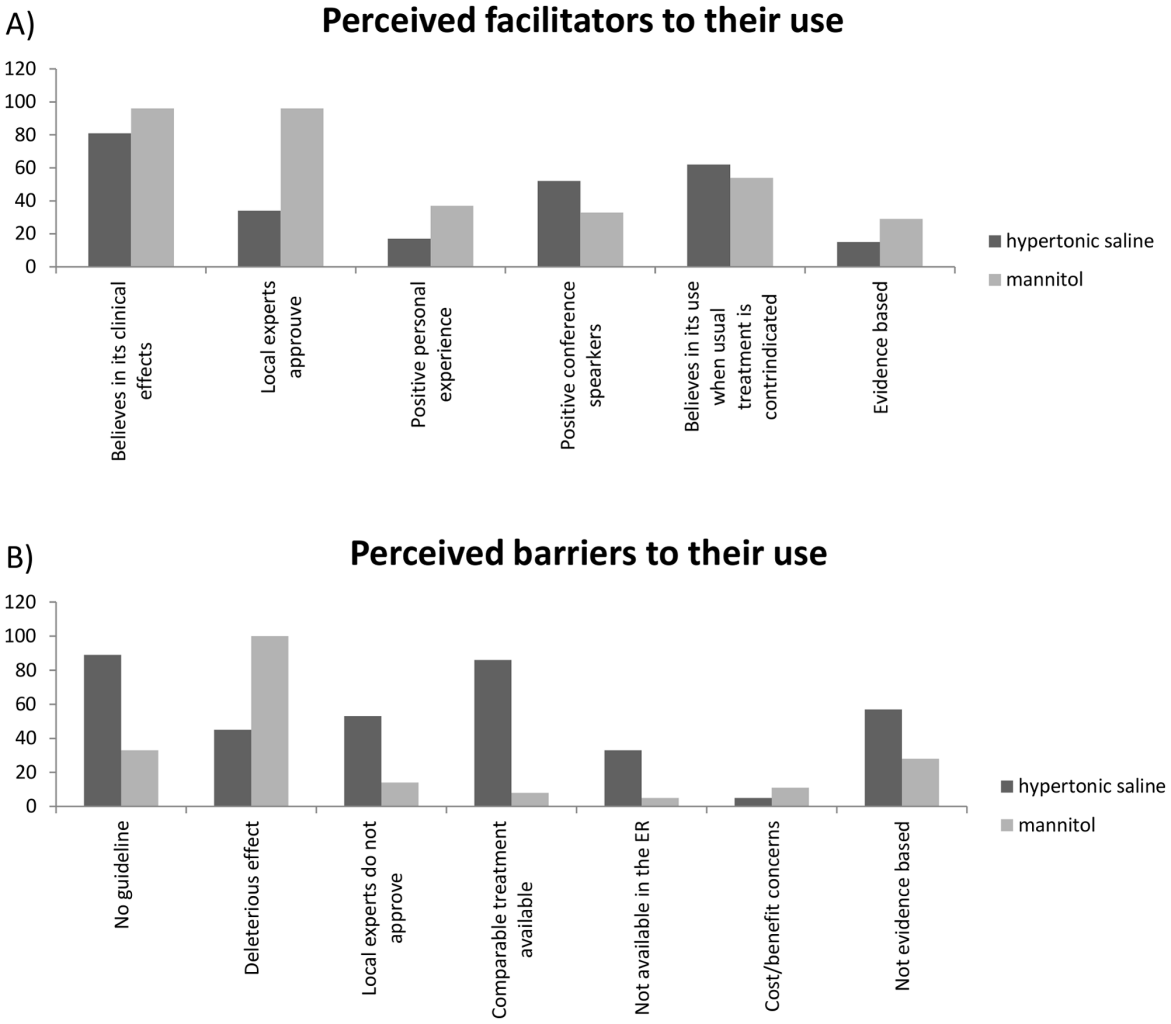
Perceived facilitators and barriers to the use of hyperosmolar solutions.

Both solutions (mannitol and hypertonic saline) are perceived to be beneficial by emergency physicians (Figure 4). For mannitol, the approval by local experts was the key facilitator (n = 96, 62%). Regarding hypertonic saline facilitators, standard treatment contraindication (n = 62, 42%) and its promotion by renowned speakers (n = 52, 35%) were the main beneficial beliefs.

#### Barriers to their use

As with the facilitators, perceived barriers differed between hypertonic saline solutions and mannitol (Figure 4). The perception that mannitol could have possible deleterious effects and could harm patients (n = 100, 68%) was the most frequently reported barrier. For hypertonic saline solutions, respondents stated that the absence of clinical guidelines approving their use (n = 89, 57%), the availability of a comparable treatment (mannitol) (n = 86, 55%) and that hypertonic saline solutions use was not evidence-based (n = 57, 36%), were the main barriers.

## Discussion

We surveyed Québec's emergency physicians involved in the management of patients with severe traumatic brain injury to understand their attitudes surrounding the use of mannitol and hypertonic saline. We observed variations in the perceived use of hyperosmolar solutions among emergency physicians, and in the reported factors potentially influencing each solution's administration. Interestingly, we observed that a significant proportion of emergency physicians reported using hypertonic saline solutions for patients with severe traumatic brain injury despite the absence of guidelines or recommendations, the main barrier to its usage.

Our study is the first to evaluate the attitudes of emergency physicians on the use of hyperosmolar solutions. Previous surveys on the utilization of hyperosmolar solutions targeted critical care physicians and neurosurgeons [2], [13], [15], and focused on hypertonic saline only. In the United Kingdom, a survey of intensive care unit directors showed that hypertonic saline was used in 50% of intensive care units to manage severe traumatic brain injury [15]. Various concentrations of saline solutions were reported to be used as an adjunct to mannitol, sometimes as a first-line therapy or as a rescue therapy to treat presumed increased intracranial pressure. The perceived frequency of use is consistent with that of our study, albeit in a different population of physicians. In a survey of Canadian neurosurgeons on the management of intracranial pressure in patients with severe traumatic brain injury, respondents agreed that hypertonic saline could be an alternative treatment, but they primarily reported using mannitol as their main therapy [13]. In a recent large retrospective cohort study conducted by our group in 6 intensive care units in Canada, we observed that one third of patients with severe traumatic brain injury received hypertonic saline during the first weeks of care [2], [14]. Our results add to the current body of literature in acute care setting by confirming the growing popularity and off-label use of hypertonic saline for the management of increased intracranial pressure in patients with severe traumatic brain injury not only in the controlled environment of the intensive care unit, but also in the emergency room.

Although the diagnosis of increased intracranial pressure is not always easy upon patient arrival in the emergency room, respondents are more prone to use hyperosmolar solutions when indirect features of presumed increased intracranial pressure were suggested in the scenario (clinical and radiological). This observation suggests that emergency physicians surveyed did not consider that hyperosmolar solutions should be used in most severe traumatic brain injury patients, but rather in a selected population where aggressive and rapid management of intracranial hypertension is required. Our study did not aim to address whether or not the use of osmolar therapy was appropriate, but rather to evaluate the use of these solutions in the pragmatic approach of care as seen in the emergency settings in trauma centers.

An increased number of studies were published over the last decade showing promising results with the use of hypertonic saline to manage increased intracranial pressure in adult patients with severe traumatic brain injury [16], [23], [24]. This likely explains the use of hypertonic saline despite the absence of strong recommendations. Unfortunately, these studies have a small sample size, typically did not evaluate clinically relevant outcomes and were performed in intensive care units or prehospital settings rather than in emergency room settings. Many editorials and narrative reviews promoting the use of hypertonic saline in this population may also have influenced emergency physicians in their practice [25], [26], [27]. However, as we observed, the emergency physicians surveyed said that evidence-based data was not the most important facilitator for the use of hyperosmolar solutions.

### Limitations and strengths

Our survey has some limitations. First, we targeted emergency physicians working in level I and II trauma centers from one Canadian province. The Québec trauma system is an organized system, in place since 1996, with well-established level I to IV trauma centers, each having specific roles in the trauma system. Results for our survey can thus be influenced by the uniform approach to trauma care in the province as compared to other regions with less organized trauma systems. Second, despite many efforts and structured methods to obtain a high response rate (web-based and paper-based questionnaires including reminders), we obtained a 50% response rate. Although this response rate could be considered moderately low in certain populations, this rate is higher than the one usually observed in surveys of emergency physicians [28]. Third, the survey was self-administered; the answers represent self-reported practice and not necessarily actual clinical practice. Our study has also several strengths starting with a rigorous methodology from the identification of the target population, the development and testing of a validated questionnaire and its administration.

### Conclusion

In our study, we observed practice variation regarding the use of hyperosmolar solutions in adult patients with severe traumatic brain injury by emergency physicians working in level I and level II trauma centers of the province of Québec, Canada. Representing around one fourth of the Canadian population, these results Despite the absence of recommendations and sufficient evidence, emergency physicians reported using hypertonic saline in specific clinical situations. The fact that half of respondents said using hypertonic saline solutions may also indicate a clinical equipoise among clinicians. These results highlight the need for better evidence-based data for the use of hyperosmolar solutions in this setting, hypertonic saline in particular. It also highlights the need to change our knowledge transfer strategies to disseminate the current level of evidence for their use. Before clinical trials comparing the use of hypertonic saline solutions with mannitol are undergone, guidelines on the management of severe traumatic brain injury should be updated following a strict evidence-based methodology such as GRADE [29].

## Supporting Information

Appendix S1 **Survey questionnaire.**
(DOCX)
