# Supplementary material for: Hyperosmolar Therapy in Severe Traumatic Brain Injury: A Survey of Emergency Physicians from a Large Canadian Province
Source: PLoS One. 2014 Apr 22;9(4):e95778. doi: 10.1371/journal.pone.0095778 (PMC3995876; doi:10.1371/journal.pone.0095778)
Supplement: Appendix S1 — Survey questionnaire. (DOCX) [file pone.0095778.s001.docx]

Appendix S1 : Survey questionnaire

## SECTION 1

Perception d’utilisation des solutions hyperosmolaires

La première question vise à évaluer votre perception d’utilisation actuelle des solutions hyperosmolaires dans le traitement de l’hypertension intracrânienne (HTIC) à la salle d’urgence chez les victimes de TCC grave.

Dans le traitement de l’hypertension intracrânienne (HTIC) à la salle d’urgence chez des victimes de traumatisme craniocérébral (TCC) grave, à quelle fréquence utilisez-vous les solutions hyperosmolaires suivantes?

|  | Jamais | Rarement | Parfois | Souvent | Toujours |
| --- | --- | --- | --- | --- | --- |
| 1.1 Solution de mannitol | 🞎 | 🞎 | 🞎 | 🞎 | 🞎 |
| 1.2 Solution de salin hypertonique | 🞎 | 🞎 | 🞎 | 🞎 | 🞎 |

## SECTION 2

Facteurs modifiant la perception d’utilisation des solutions hyperosmolaires.

Les questions suivantes visent à évaluer votre pratique actuelle en ce qui a trait à l’utilisation de solutions hyperosmolaires (mannitol, salin hypertonique ou autre) pour le traitement de l’HTIC chez les victimes de TCC grave.

Quel est le pourcentage approximatif de victimes de TCC grave présentant les caractéristiques suivantes chez qui VOUS allez utiliser une solution hyperosmolaire?

|  | Jamais (0% des patients) | Rarement (1% à 10% des patients) | Parfois (11% à 25% des patients) | Souvent (de 26 à 50% des patients) | Habituellement (de 51% à 75% des patients) | Presque toujours (de 76 à 99% des patients) | Toujours (100% des patients) |
| --- | --- | --- | --- | --- | --- | --- | --- |
| **2.1** Patient de 36 ans atteint d’un TCC grave contondant, sans autre traumatisme associé, avec SV normaux, présentant un GCS à 3 (V=1, Y=1, M=1) | 🞎 | 🞎 | 🞎 | 🞎 | 🞎 | 🞎 | 🞎 |
| **2.2** Même patient qu’en 1, mais présentant une mydriase fixe bilatérale non réactive à l’arrivée des techniciens ambulanciers sur le site de l’accident | 🞎 | 🞎 | 🞎 | 🞎 | 🞎 | 🞎 | 🞎 |
| **2.3** Même patient qu’en 1, mais avec un GCS à 6 (V=1, Y=1, M=4) | 🞎 | 🞎 | 🞎 | 🞎 | 🞎 | 🞎 | 🞎 |
| **2.4** Même patient qu’en 1, mais âgé de 60 ans | 🞎 | 🞎 | 🞎 | 🞎 | 🞎 | 🞎 | 🞎 |
| **2.5** Même patient qu’en 1, mais présentant une certaine instabilité hémodynamique | 🞎 | 🞎 | 🞎 | 🞎 | 🞎 | 🞎 | 🞎 |
| **2.6** Même patient qu’en 1, mais présentant une mydriase fixe réactive | 🞎 | 🞎 | 🞎 | 🞎 | 🞎 | 🞎 | 🞎 |
| **2.7** Même patient qu’en 1, mais connu pour des comorbidités importantes | 🞎 | 🞎 | 🞎 | 🞎 | 🞎 | 🞎 | 🞎 |
| **2.8** Même patient qu’en 1, mais avec une déviation de la ligne médiane de > 8 mm à la TDM cérébrale | 🞎 | 🞎 | 🞎 | 🞎 | 🞎 | 🞎 | 🞎 |
| **2.9** Même patient qu’en 1, mais avec une compression des citernes de la base à la TDM cérébrale | 🞎 | 🞎 | 🞎 | 🞎 | 🞎 | 🞎 | 🞎 |
| **2.10** Même patient qu’en 1, mais démontrant un œdème diffus à la TDM cérébrale | 🞎 | 🞎 | 🞎 | 🞎 | 🞎 | 🞎 | 🞎 |
| **2.11** Même patient qu’en 1, mais sans anomalie à la TDM cérébrale | 🞎 | 🞎 | 🞎 | 🞎 | 🞎 | 🞎 | 🞎 |

## SECTION 3

Les questions suivantes visent à donner un aperçu des obstacles potentiels et des données probantes pouvant influencer l’utilisation des solutions de mannitol et de salin hypertonique dans le traitement des victimes de TCC grave.

### 3.1 OBSTACLES À L’UTILISATION DE SOLUTIONS DE SALIN HYPERTONIQUE

3.1.1 Veuillez sélectionner le ou les facteur(s) qui, selon vous, constitue(nt) un OBSTACLE à l’utilisation de la solution de salin hypertonique dans le traitement de l’HTIC chez les victimes de TCC grave?

| 3.1.1.1 Il n’existe aucun guide de pratique appuyant son utilisation | 🞎 |
| --- | --- |
| 3.1.1.2 Vous craignez que les effets secondaires potentiels l’emportent sur les effets bénéfiques potentiels du traitement | 🞎 |
| 3 Vos experts locaux n’appuient pas l’usage de SSH | 🞎 |
| 4 Un traitement comparable (mannitol) est déjà largement répandu | 🞎 |
| 5 La SSH n’est pas rapidement disponible à la salle d’urgence | 🞎 |
| 6 Vous avez des préoccupations en ce qui a trait au coût/bénéfice de la SSH | 🞎 |
| 7 Son usage n’est pas basé sur des données probantes | 🞎 |

3.1.2 Si vous avez choisi « son usage n’est pas basé sur des données probantes », quels aspects des données probantes actuelles constituent des OBSTACLES à l’usage de la SSH? (Cochez toutes les réponses pertinentes)

| 3.1.2.1 Nombre insuffisant d’essais cliniques randomisés contrôlés à ce jour | 🞎 |
| --- | --- |
| 2 Études biaisées probables en raison de problèmes de méthodologie | 🞎 |
| 3 Résultats divergents entre les différentes études | 🞎 |
| 4 Bienfaits cliniques insuffisants | 🞎 |
| 5 Estimation imprécise des bienfaits cliniques potentiels | 🞎 |
| 6 Estimation imprécise des effets secondaires potentiels | 🞎 |
| 7 Autre (Veuillez préciser) | 🞎 |

### 3.2 FACTEURS FACILITATEURS À L’UTILISATION DE SOLUTIONS DE SALIN HYPERTONIQUE

3.2.1 Veuillez sélectionner le ou les facteur(s) qui, selon vous, APPUIENT l’utilisation de la solution de salin hypertonique dans le traitement de l’HTIC chez les victimes de TCC grave?

| 3.2.1.1 Vous croyez que les effets bénéfiques potentiels liés au traitement l’emportent sur les effets secondaires possibles | 🞎 |
| --- | --- |
| 2 Vos experts locaux appuient l’usage de la SSH au sein de cette population de patients | 🞎 |
| 3 Vous avez vécu une expérience personnelle concluante avec ce traitement | 🞎 |
| 4 Vous avez entendu des témoignage(s) concluant présenté(s) lors de congrès | 🞎 |
| 5 Vous croyez à son utilisation lors de la présence de contre-indications du traitement usuel | 🞎 |
| 6 Son utilisation est basée sur des données probantes | 🞎 |

3.2.2 Si vous avez choisi « son utilisation est basée sur des données probantes », quels aspects des données probantes actuelles APPUIENT l’utilisation de la SSH ? (Cochez toutes les réponses pertinentes)

| 3.2.2.1 Nombre suffisant d’essais cliniques randomisés contrôlés à ce jour | 🞎 |
| --- | --- |
| 2 Études non biaisées probables en raison de la bonne qualité méthodologique | 🞎 |
| 3 Résultats convergents et comparables entre les différentes études | 🞎 |
| 4 Bienfaits cliniques importants | 🞎 |
| 5 Estimation précise des bienfaits cliniques potentiels | 🞎 |
| 6 Estimation précise des effets secondaires potentiels | 🞎 |
| 7 Autre (Veuillez préciser) | 🞎 |

3.3 OBSTACLES À L’UTILISATION DE SOLUTIONS DE MANNITOL

3.3.1 Veuillez sélectionner le ou les facteur(s) qui, selon vous, constitue(nt) un OBSTACLE à l’utilisation de la solution de mannitol dans le traitement de l’HTIC chez les victimes de TCC grave? (Cochez toutes les réponses pertinentes)

| 3.3.1.1 Il n’existe aucun guide de pratique appuyant son utilisation | 🞎 |
| --- | --- |
| 2 Vous craignez que les effets secondaires potentiels l’emportent sur les effets bénéfiques potentiels du traitement | 🞎 |
| 3 L’administration de la solution de mannitol augmente la charge de travail des médecins/infirmières de façon considérable | 🞎 |
| 4 La solution de mannitol n’est pas rapidement disponible au département d’urgence | 🞎 |
| 5 Vous avez des préoccupations en ce qui a trait au coût/bénéfice de la solution de mannitol | 🞎 |
| 6 Son usage n’est pas basé sur des données probantes | 🞎 |

3.3.2 Si vous avez choisi « son usage n’est pas basé sur des données probantes », quels aspects des données probantes actuelles constituent des OBSTACLES à l’usage de la solution de mannitol? (Cochez toutes les réponses pertinentes)

| 3.3.2.1 Nombre insuffisant d’essais cliniques randomisés contrôlés à ce jour | 🞎 |
| --- | --- |
| 2 Études biaisées probables en raison de problèmes de méthodologie | 🞎 |
| 3 Résultats divergents entre les différentes études | 🞎 |
| 4 Bienfaits cliniques insuffisants | 🞎 |
| 5 Estimation imprécise des bienfaits cliniques potentiels | 🞎 |
| 6 Estimation imprécise des effets secondaires potentiels | 🞎 |
| 7 Autre (Veuillez préciser) | 🞎 |

### 3.4 FACTEURS FACILITATEURS À L’UTILISATION DE SOLUTIONS DE MANNITOL

3.4.1 Veuillez sélectionner le ou les facteur(s) qui, selon vous, APPUIENT l’utilisation de la solution de mannitol dans le traitement de l’HTIC chez la victime de TCC grave? (Cochez toutes les réponses pertinentes)

| 3.4.1.1 Vous croyez que les effets bénéfiques potentiels liés au traitement l’emportent sur les effets secondaires possibles | 🞎 |
| --- | --- |
| 2 Vos experts locaux appuient l’usage de la solution de mannitol au sein de cette population de patients | 🞎 |
| 3 Vous avez vécu une expérience personnelle concluante avec ce traitement | 🞎 |
| 4 Vous avez entendu des témoignage(s) concluant présenté(s) lors de congrès | 🞎 |
| 5 L’administration de la solution de mannitol n’augmente pas la charge de travail des médecins/infirmières de façon considérable | 🞎 |
| 6 Son utilisation est basée sur des données probantes | 🞎 |

3.4.2 Si vous avez choisi « son utilisation est basée sur des données probantes », quels aspects des données probantes actuelles APPUIENT l’utilisation de solutions de mannitol? (Cochez toutes les réponses pertinentes)

| 3.4.2.1 Nombre suffisant d’essais cliniques randomisés contrôlés à ce jour | 🞎 |
| --- | --- |
| 2 Études non biaisées probables en raison de la bonne qualité méthodologique | 🞎 |
| 3 Résultats convergents et comparables entre les différentes études | 🞎 |
| 4 Bienfaits cliniques importants | 🞎 |
| 5 Estimation précise des bienfaits cliniques potentiels | 🞎 |
| 6 Estimation précise des effets secondaires potentiels | 🞎 |
| 7 Autre (Veuillez préciser) | 🞎 |

## SECTION 4

Données démographiques

Cette dernière section a trait aux caractéristiques démographiques des répondants. Une fois de plus, toutes les réponses sont entièrement anonymes.

1. 4.1 Quelle est votre formation de base?

| Médecine familiale | 🞎 |
| --- | --- |
| Médecine familiale avec formation complémentaire en médecine d’urgence CCFMU | 🞎 |
| Spécialité en médecine d’urgence | 🞎 |

1. 4.2 Depuis combien d’années êtes-vous en pratique clinique

| Moins d’un an | 🞎 |
| --- | --- |
| De 1 à 5 ans | 🞎 |
| De 6 à 10 ans | 🞎 |
| De 11 à 20 ans | 🞎 |
| Plus de 20 ans | 🞎 |

1. 4.3 Dans quel type de milieu pratiquez-vous?

| Centre tertiaire de traumatologie | 🞎 |
| --- | --- |
| Centre secondaire régional de traumatologie | 🞎 |
| Centre secondaire de traumatologie | 🞎 |

1. 4.4.1Y a-t-il un neurochirurgien dans votre milieu?

| Oui | 🞎 |
| --- | --- |
| Non | 🞎 |

4.4.2 Si non, à quelle distance se trouvent le centre hospitalier avec une équipe neurochirurgicale?

| Moins de 50 km | 🞎 |
| --- | --- |
| 51-100 km | 🞎 |
| Plus de 100 km | 🞎 |

1. 4.5 Combien de quarts de travail faites-vous à la salle d’urgence par mois?

| Moins de 4 | 🞎 |
| --- | --- |
| Entre 5 et 8 | 🞎 |
| Entre 9 et 11 | 🞎 |
| Plus de 12 | 🞎 |

1. 4.6 Combien voyez-vous de victimes de TCC grave (GCS ≤8) par année?

| Moins de 3 | 🞎 |
| --- | --- |
| Entre 4 et 7 | 🞎 |
| Entre 8 et 10 | 🞎 |
| Plus de 10 | 🞎 |
